# Biomaterials for Testicular Bioengineering: How far have we come and where do we have to go?

**DOI:** 10.3389/fendo.2023.1085872

**Published:** 2023-03-16

**Authors:** Bianca de Oliveira Horvath-Pereira, Gustavo Henrique Doná Rodrigues Almeida, Leandro Norberto da Silva Júnior, Pedro Gabriel do Nascimento, Bárbara de Oliveira Horvath Pereira, João Victor Barbosa Tenório Fireman, Maria Laura dos Reis Ferre Pereira, Ana Claudia Oliveira Carreira, Maria Angelica Miglino

**Affiliations:** ^1^ Department of Surgery, School of Veterinary Medicine and Animal Science, University of São Paulo, São Paulo, Brazil; ^2^ Centre for Natural and Human Sciences, Federal University of ABC, São Paulo, Brazil

**Keywords:** testis, biomaterials, spermatogenesis, bioengeneering, reproduction

## Abstract

Traditional therapeutic interventions aim to restore male fertile potential or preserve sperm viability in severe cases, such as semen cryopreservation, testicular tissue, germ cell transplantation and testicular graft. However, these techniques demonstrate several methodological, clinical, and biological limitations, that impact in their results. In this scenario, reproductive medicine has sought biotechnological alternatives applied for infertility treatment, or to improve gamete preservation and thus increase reproductive rates *in vitro* and *in vivo*. One of the main approaches employed is the biomimetic testicular tissue reconstruction, which uses tissue-engineering principles and methodologies. This strategy pursues to mimic the testicular microenvironment, simulating physiological conditions. Such approach allows male gametes maintenance in culture or produce viable grafts that can be transplanted and restore reproductive functions. In this context, the application of several biomaterials have been proposed to be used in artificial biological systems. From synthetic polymers to decellularized matrixes, each biomaterial has advantages and disadvantages regarding its application in cell culture and tissue reconstruction. Therefore, the present review aims to list the progress that has been made and the continued challenges facing testicular regenerative medicine and the preservation of male reproductive capacity, based on the development of tissue bioengineering approaches for testicular tissue microenvironment reconstruction.

## 1 Introduction

Recently, a worldwide decline of male fertility parameters has been observed, in both humans and animals. In humans, almost 50% of infertile couples have the male component as the major cause (1–3). Regarding other species, fertility preservation of endangered species has been the main goal, and the understanding of their reproductive biology assists on their conservation and management (4, 5).

Several factors may influence male fertility; however, there is no specific etiology for almost 40% of infertile men (6–8). Male infertility may occur due to several conditions, which include hormonal deficits (9), anatomical or genetic abnormalities (10), systemic illnesses, infections, traumas, intoxications, autoimmune diseases, environmental exposure or even lifestyle (11–13). Malignant testicular neoplasms are one of the greatest causes of testicular tissue degeneration, being the highest incident type of cancer in men, also having a high incidence in domestic species as dogs (14, 15). These neoplasms may occur due to hereditary mutations, but the greater percentage comes from environmental factors (16–20).

Considering that genome reprogramming steps take place during gametogenesis and early development, abnormal genome epigenetic reprogramming is highlighted as a contributing factor for male infertility (21, 22). Epigenetic processes are defined as hereditary alteration that affect gene expression, not modifying the DNA sequence (23). Among these alterations, there are DNA methylations, histones alterations and non-coding RNAs synthesis, which can be transmitted to the offspring (24, 25).

Facing this scenario, advances in male fertility preservation may guarantee the reestablishment of reproductive functions or germ cells safeguarding through the development of reproductive biotechnologies (26). One of the greatest achievements was the application of assisted reproduction technologies (ART) for recent generations of humans and the increase of livestock production (27, 28). More recently, due to environmental sustainability policies, such technologies have been devoted to endangered species conservation, seeking not only to maintain the current population, but to also preserve the genetic heritage (5, 29, 30).

Some of the most prominent approaches to preserve male fertility include sperm and testicular tissue cryopreservation; germ cells transplantation, and testicular grafts (31–34). An efficient germ cell maintenance is essential for *in vitro* fertilization as for intracytoplasmic sperm injection (ICSI), which is one of the most well-succeeded techniques in assisted reproduction (35, 36). When the spermatozoid production is not possible, the use of spermatogonial stem cells (SSCs) is considered a viable option (35), but due to several experimental difficulties as stem cells isolation, identification, purification and *in vitro* maintenance, this approach remains limited (36). Although its broad potential in the reproduction field, its effectiveness was reported in rodents, remaining a challenge in larger species (37, 38).

Essential processes of male reproductive physiology as spermatogenesis and maintenance of spermatic viability are highly dependent of the molecular microenvironment (39). Among the other elements that compound the tissue microenvironment as growth factors, hormones and other biofactors, the extracellular matrix (ECM) is highlighted due to its role in testicular tissue homeostasis (40, 41). ECM also provides three-dimensionality, which enhances the interaction between the cell and the extracellular environment, increasing cell susceptibility to molecular signaling from the ECM and other exogenous factors (42).

A promising approach both for degenerated testicular tissue replacement and for *in vitro* germ cells maintenance is the development of biomimetic testicular tissues that contain similar morphophysiological characteristics to those found *in vivo* (40). In this scenario, some bioengineering strategies that associate biomaterials, cells and bioactive factors have been proposed to provide greater complexity to the artificial tissues (41).

A biomaterial considered eligible to be applied in a biomimetic system, must have several physico-chemical properties as, suitable morphology, mechanic resistance, and porous structure (43–45). It must also be biocompatible and have an acceptable biodegradability that allows the interaction with cells (43–45). Synthetic biomaterials as polyesters and polyprolactone are viable alternatives to produce three-dimensional scaffolds that may be chemically altered to adapt for diverse contexts (39). However, due to their weak interaction with cell membrane adhesion proteins, such polymers are not able to fully mimic the ECM biological properties (39). Natural polymers, otherwise, due to their biological origin, present better cytocompatibility, which allows the development of a more reliable microenvironment, however, such components do not provide the entire ECM complexity (46). A more complex alternative to all these biomaterials is the application of decellularized matrixes, which, if well preserved, contain the main fibrillary and non-fibrillary components (4, 5, 47).

Therefore, this review aimed to describe the advances and challenges of tissue engineering for testicular tissue reconstruction and the development of artificial *in vitro* systems that can preserve and develop male germ cells, highlighting their advantages over the main methods of male fertility preservation. Furthermore, this article aims to discuss how bioengineering can be an important and innovative approach to andrological regenerative medicine, highlighting the role of the testicular microenvironment as a protagonist in the reproductive potential maintenance of both humans and other species.

## 2 Testicular morphophysiology

### 2.1 Testicular architecture, structure and ultrastructure

Anatomically, male reproductive tract is constituted by testis, epididymis, vas deferens, urethral adnexal glands (ampullae, vesicular glands, prostate, and bulbourethral gland) and the penis (48). The testis are paired organs located outside the abdominal cavity inside the scrotum. The testicular surface is covered by the tunica albuginea, with a thickness of 1 to 2 mm, composed of collagen fibers and containing the blood vessels (testicular artery and testicular vein) that are visible on the testicular surface, in which each species present a characteristic pattern (49, 50). Regarding muscle constitution, the testis is covered by the smooth muscle dartos tunic and suspended by the cremaster muscle, which contribute to testicular thermoregulation, moving the gonads away or closer to the inguinal-abdominal area (51). This mechanism is related to testicular temperature maintenance, which is essential for spermatogenesis to occur normally. In species that the testis are inside the scrotum, the temperature of the gonads must be between 4° and 7° C below body temperature (51, 52).

**Figure 1:**
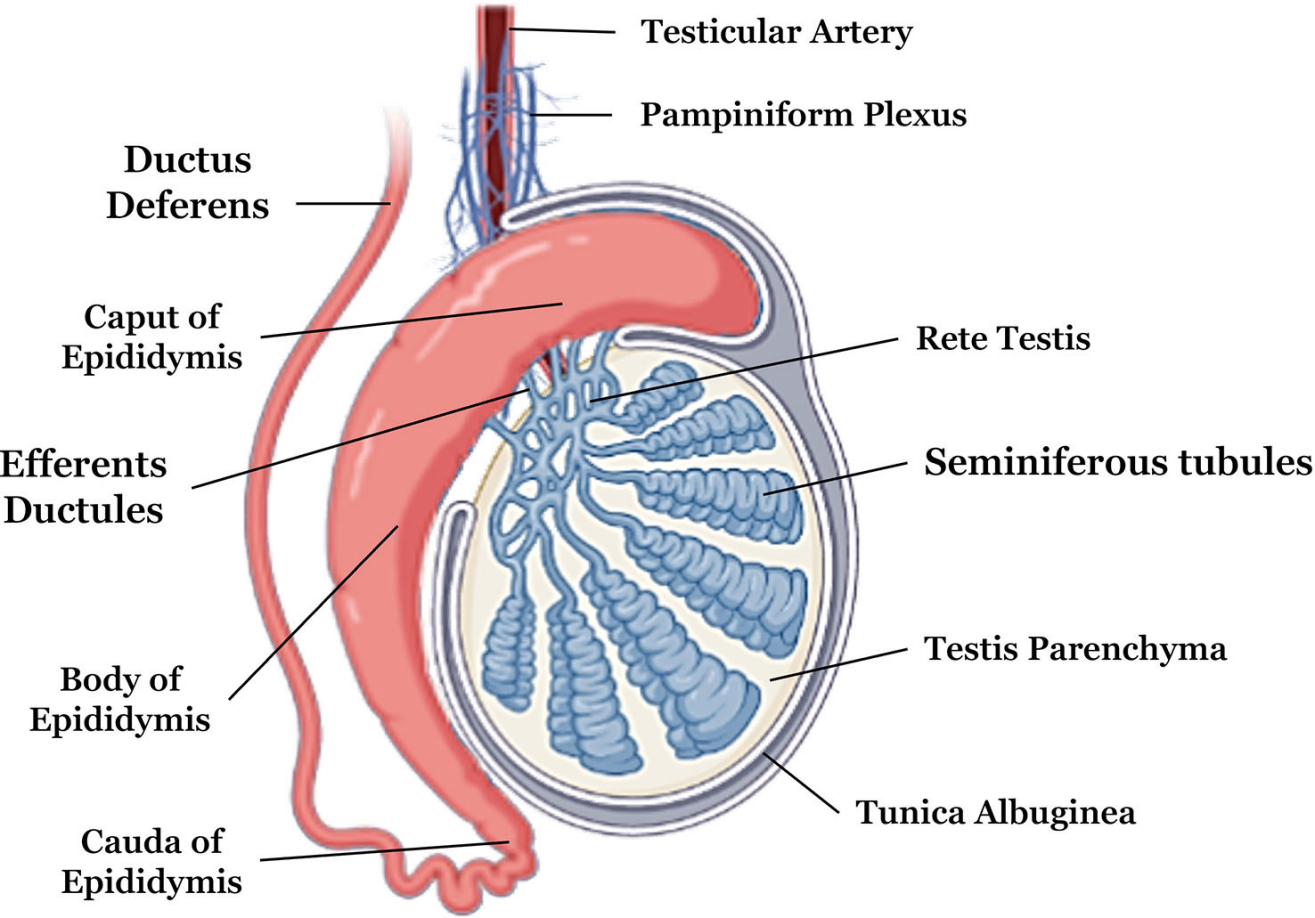
Anatomical description of testicular and epididymal structures. Adapted from Biorender .

More externally, the testis are composed of a fibrous capsule called the tunica albuginea; more internally, there are the septa and the mediastinum, which make up the connective tissue (53). The tunica albuginea gives off the septa that spreads into the testicle. As the septa enters the testis, the testicular parenchyma divides into pyramidal lobes (54). These septa tend towards the central region forming the mediastinum of the testis, and their location can vary from axial to displaced towards the epididymis (55) (**Figure 1**).

Histologically, the testicular tissue has several cell types, which can be divided in germ cells lineage, from gonocytes to mature spermatozoids, support, and immune cells (56). The extracellular matrix is an association of structural and functional components that provide not only a structural network for cells but also a biochemical and biomechanical signaling that is able to impact on cell physiology directly (57, 58). The testicular ECM is formed by several components as collagen fibers, elastic fibers, glycosaminoglycans and proteoglycans (56) (**Table 1**).

**Table 1:** Cellular and extracellular components of testicular and epididymal tissues.

Type of Cell	Extracellular Matrix	References
Fibroblasts Macrophages Leydig cells Peritubular myoblasts Endothelial cells Sertoli cells Smooth muscle cells Gonocytes Spermatogonial stem cells Primary spermatocytes Secondary spermatocytes Spermatids Spermatozoids	Type I, III e IV collagen Elastic fibers Laminin Glycosaminoglycans Proteoglycans Fibronectin	(48, 59–63)

Functionally, testicular ECM is organized into a specialized structure called the blood-testis barrier (BTB), formed mainly by type IV collagen, laminin, heparan sulfate proteoglycans, entactin and fibronectin (64). This barrier, along with the tight junctions of Sertoli cell membranes, restricts the flow of molecules from the bloodstream, selecting which components will come into the testicular parenchyma (65, 66).

In mammals, the testis consist of two compartments: the interstitial and seminiferous tubules compartments (67). The interstitial one contains nerves, blood, and lymphatic vessels (67). Its main cell type is the Leydig cells, that are responsible for testosterone synthesis. Other important cell types presented in this region are peritubular, endothelial, smooth muscle, perivascular cells, and testicular macrophages (68). Regarding the seminiferous tubules, several cell layers constitute them. In the periphery, there are Sertoli cells and spermatogonium. Inside the tubules, there are spermatic cells lineage, which includes spermatocyte I, spermatids and more centrally, spermatozoids (69).

The testicular microenvironment is essential for the spermatogenesis process to occur normally, providing favorable conditions for anchorage, cell growth, nutrient diffusion, and mechanical support necessary for tissue homeostasis (56). The complex structure of the testis directly acts on sperm maturation and the male hormones production (70). Sertoli cells are the main cell type responsible for the production and secretion of metal ion binding proteins, lipids, proteases, protease inhibitors, hormones, and growth factors. This secretion products act on germ cells, tissue remodeling, spermatid release, basement membrane formation and intercellular junctions (65). Another cell type essential for spermatogenesis is the Leydig cell, which acts directly on Sertoli cells and sperm development (65). Hormones such as testosterone and follicle-stimulating hormone (FSH) act on Sertoli cells gene expression, regulating their activity according to the spermatogenesis cycles (71).

Another important structure that is connected to the testicles is the epididymis, which is divided into caput, body, and cauda, situated longitudinally in the caudal portion of the testis (72). The caput of the epididymis is in the upper portion, while the cauda is located in the lower portion of the testis. The epididymis consists of a long tube that is coiled. In the caput the sperm maturation phase occurs, and in the body and cauda, sperm motility occurs (73, 74).

### 2.2 Spermatogenesis

**Figure 2:**
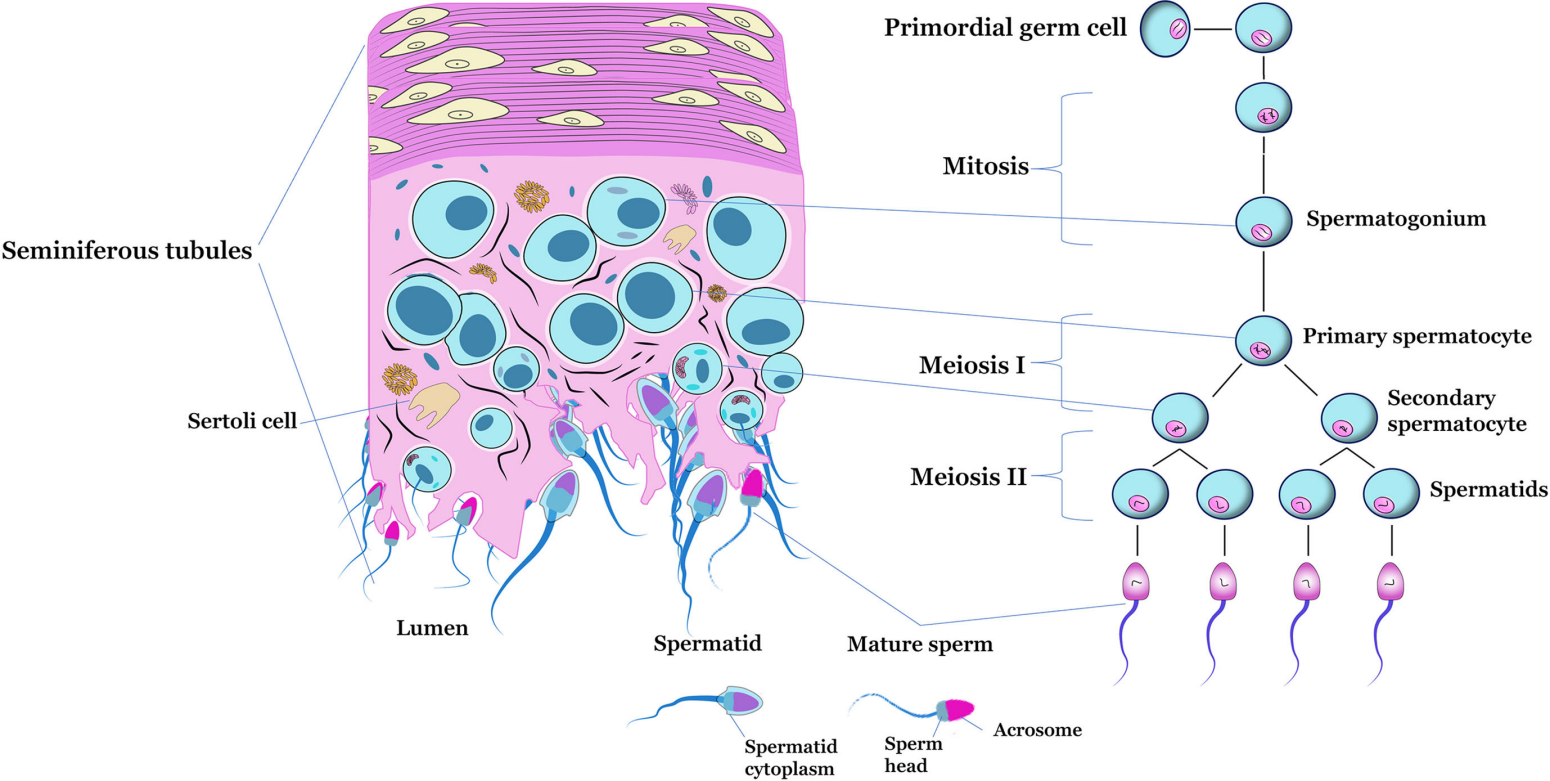
Schematic figure of a transverse section of the seminiferous tubule, highlighting the main cell types located inside (left). Representation of the of spermatogenesis (right).

The development of male gametes is a complex differentiation process that takes place in the testis and produces sperm (75, 76). Spermatogenesis begins with the proliferation and differentiation of diploid spermatogonial stem cells, followed by meiosis of spermatocytes that form round spermatids (77). In mammals, the spermatogenesis process is composed of three distinct phases: the mitotic or spermatogonial phase, in which the gonocytes or stem spermatogonia undergo mitotic divisions until the formation of primary spermatocytes; meiotic or spermatocyte phase, stage in which spermatocytes undergo reduction divisions that result in the formation of spermatids; and the spermiogenesis or differentiation phase, the period in which the morphological and functional changes of spermatids occur until the moment of their release into the lumen of the seminiferous tubule, where they become spermatozoa (38, 78, 79). The stages of differentiation of primordial germ cells into mature sperm are schematized in **Figure 2**.

During embryonic development, primordial germ cells migrate from the yolk sac region to the undifferentiated gonads (80). After reaching the forming gonad, primordial cells begin the process of division before forming gonocytes. In males, these gonocytes undergo differentiation before puberty to form A0 spermatogonia, from which other germ cells originate (81). During the first phase of spermatogenesis, the proliferation and differentiation of spermatogonia occurs (75). The types of undifferentiated and differentiated spermatogonia vary according to the species (82).

In mice and rats, spermatogonia are divided into three types: A, Intermediate and B. Type A spermatogonia are subdivided, according to morphological criteria, into A isolated (Ai), A paired (Ap), A aligned (Aal), A1, A2, A3 and A4 (83). This classification varies according to the degree of differentiation, with Ai spermatogonia being the least differentiated and A4 the most differentiated within the type A subdivision. (84). Ap and Aal spermatogonia are called proliferative spermatogonia, A1 to A4 spermatogonia as in differentiation, Intermediate and B spermatogonia as differentiated (85). In humans, spermatogonia divide into dark type (A_dark_), pale type (A_pale_) and type B. Type A_dark_ spermatogonia reproduce through mitosis that generate both A_dark_ and A_pale_ spermatogonia (81). During adult life, undifferentiated A_pale_ spermatogonia divide, giving rise to B spermatogonia. A_dark_ spermatogonia are quiescent reserve cells, as they show low proliferative capacity throughout spermatogenic activity, while A_pale_ spermatogonia are in continuous proliferation (86). Although both types of spermatogonia (A_dark_ and A_pale_) are in the basement membrane, they differ morphologically, with A_dark_ being small, round, or slightly ovoid, while A_pale_ are larger, oval or nearly round (87).

The period of spermatogenesis varies according to the species. In cattle, for example, the entire spermatogenesis process lasts an average of 61 days, divided into three phases (33). The initial phase, known as spermatocytogenesis, is the process by which germ cells undergo mitotic divisions, and after the first division there are germ stem cells (type A spermatogonia) and primary spermatocytes (from type B spermatogonia). To give rise to primary spermatocytes, the mitotic division of A1 spermatogonia into differentiated cells called A2, A3, intermediate, B1 and B2 spermatogonia must occur (88). During the second phase, there is a reduction in the number of chromosomes, originating a haploid cell, carrying out the recombination and segregation of the genetic material. In this way, the primary spermatocytes resulting from the first phase begin DNA replication, later entering the first meiotic division to produce spermatocytes. Secondary, such spermatocytes rapidly enter the second meiotic division resulting in rounded haploid spermatids (89, 90).

During the third phase, called spermiogenesis, no more cell division phases occur, so during this phase, spermatids suffer morphological changes, differentiating into spermatozoa (91). These changes include the formation and development of the acrosome and flagellum, chromatin condensation, remodeling, and elongation of the nucleus. Besides that, there is a cytoplasm removal, which occurs before the spermatid release during the spermiation, a phase characterized for the spermatozoa releasing into the seminiferous tubules lumen (92, 93). However, these spermatozoa do not have the ability to fertilize the oocyte, and it is necessary for these spermatozoa to pass into the straight tubules, reach the rete testis and reach the epididymis, where they start the maturation process (94, 95). Thus, in bulls the mitotic division phase lasts 21 days, the meiotic division phase 23 days and spermiogenesis 17 days, resulting in 61 days of spermatogenesis (88), and in humans and rats, the spermatogenesis process lasts 74 and 35 days, respectively (96, 97).

Spermatogenesis is also regulated by endocrine factors depending on the activity of the hypothalamic-pituitary-testicular axis, whereby Gonadotropin Releasing Hormone (GnRH) stimulates the anterior pituitary to release Luteinizing Hormone (LH) and Follicle Stimulating Hormone (FSH), that will produce stimuli to produce gonadal steroids and for the development of germ cells (98, 99). LH hormone receptors (LHR) stimulate the process of steroidogenesis and act during the development and maintenance of spermatogenesis (100), their expression takes place in Leydig cells and are essential for fertility in mammals (68).

During spermatogenesis, three hormones are essential for the process to occur without modification, namely testosterone, FSH and LH. The lack of any of these hormones cause germ cell apoptosis, and when administered, these hormones suppress apoptosis (101). Therefore, germ cells require the presence of these hormones for their survival. When there is a lack of testosterone, round spermatids do not complete the transition phase to elongated spermatids, as there is a loss of spermatids binding to Sertoli cells (102). FSH acts indirectly through Sertoli cells, being associated with early stages of spermatogenesis, especially during spermatocytogenesis and meiosis (103). On the other hand, testosterone is present in the later stages of spermatogenesis, such as in the spermatid differentiation stage and potentiating the effect of FSH (104).

In cattle, Leydig cells acquire the ability to respond to the LH stimulus at puberty (105), which begin to produce increasing amounts of testosterone, exerting control over Sertoli cell differentiation and, consequently, cell growth (101). Therefore, any interruption or alteration that occurs during the spermatogenesis process can generate changes in the pattern of cell development, affecting the reproductive capacity of animals (106). Testicular hormone regulation is summarized in **Table 2**.

**Table 2:** Hormones involved in testicular physiology.

Hormone	Type of Hormone	Biological Role	Reference
**Gonadotropin Releasing Hormone (GnRH)**	Trophic peptide	GnRH is secreted in the hypothalamus and is responsible for inducing the release of FSH and LH by the anterior pituitary according to the frequency of the secretion pulse. Low frequency pulses of GnRH stimulate FSH release and high frequency pulses stimulate LH release.	(66)
**Follicle Stimulating Hormone (FSH)**	Gonadotropin	FSH is secreted in the anterior pituitary. It acts on Sertoli cells modulating their gene expression.	(65)
**Testosterone**	Steroid	Secreted by Leydig cells in the testicular parenchyma acting on receptors on Sertoli cells modulating their gene expression. It also acts in the maintenance of BTB and the adhesion of spermatids in the testis.	(66)
**Luteinizing Hormone (LH)**	Gonadotropin	Synthesized in the anterior pituitary. Acts on Leydig cells stimulating the production of Testosterone	(66)

## 3 Current strategies to reestablish testicular functionality

### 3.1 Testicular tissue cryopreservation

Testicular tissue cryopreservation is one of the alternatives to preserve human and animal fertility. Protection of male fertility can be performed by several techniques such as progenitor cells cryopreservation, testicular stem cells cryopreservation (spermatogonic stem cells or SSCs) or cryopreservation of testicular tissue fragments (107). The freezing of semen is the standard and most used technique for the preservation of male fertility in men and animals, being routinely performed in clinics and farms (108, 109). However, this technique is unfeasible when dealing with juvenile and prepubertal individuals, whose gonads have not yet started to produce sperm, or in adults in which mature sperm is not produced due to the occurrence of pathological and genetic disorders (110).

In such cases, testicular tissue cryopreservation is one of the viable alternatives (111, 112). The testis has a high number of germ cells, especially spermatogenic cells, which can offer an unlimited number of male gametes, when properly cultivated and preserved (33). This technique is used as treatment for several types of cancers, which mainly affect the spermatogenesis niche, and induce the death of spermatogonial stem cells (SSCs), reducing the sperm count in men (82). In addition to that, aiming to seek endangered species conservation, several studies have been carried out using this technique to try to preserve male reproductive tissue samples to further uses (32).

Studies have demonstrated that methods of collection and preservation of testis from sexually immature individuals, and from adult animals, alive or *postmortem* (113, 114). After immediate tissue recovery or cryopreservation, the fragments can be cultured *in vivo* or *in vitro* to obtain viable sperm (115). That is, the cryopreservation of testicular tissue fragments is used to preserve the fertility of prematurely dead animals, as well as those undergoing treatments that cause infertility, such as cancer treatment. The spermatogenic cells, present in the testicular fragments, can resume their functions *in vitro* after thawing, the genetic resources of high-value animals and the preservation of endangered species (116, 117).

Studies using both animal and human tissues generally advocate a DMSO-based cryopreservation medium for immature tissue cryopreservation and a glycerol-based medium for mature testicular tissues (35, 112, 118). Testicular tissue cryopreservation can be performed using techniques such as slow freezing, fast freezing, and vitrification; however, protocols for using these techniques are still being tested in different species (112, 119, 120).

Slow freezing is performed using a machine that gradually reduces the temperature until the frozen state of the testicular fragments is reached. The fragments are initially exposed to an equilibrium solution containing cryoprotective agent (CPAs) at 4° for 10 to 15 minutes, after which they are transferred to cryogenic flasks and stored in liquid nitrogen (121). This method allows the tissue to be less exposed to the deleterious effects of CPAs, however there is a high possibility of crystal formation during the process, which may invalidate the use of tissue after thawing (122). It is mostly used in immature human tissues and is associated with the survival of spermatogonia (35).

Vitrification is a technique widely used in the cryopreservation of female gonadal tissue, and in the research of this technique for the cryopreservation of testicular tissue, the technique used was solid surface vitrification. This technique consists of exposing the fragments to a vitrification solution, after which the fragments are placed in a metal cube above the liquid nitrogen, which allows the tissue to be cooled in an ultra-rapid way and, after freezing, they are stored in cryotubes and maintained in liquid nitrogen (123). Among the cryopreservation techniques, the most common methods are slow freezing and vitrification. Vitrification has the lowest operating cost and is easy to perform, in addition to avoiding crystallization in a more effective way than the other techniques due to the ultra-fast cooling (124).

During the freezing and thawing processes of the material, the loss of spermatogonia is inevitable, and to improve cell survival, cryoinjury caused by the formation of intracellular ice crystals must be avoided through the addition of cryoprotective agent and the control of freezing and thawing rates. Testicular cryopreservation is an economical and efficient method to preserve genetic material; however, its techniques are still being tested and improved, so there are several approaches that involve numerous positive and negative points to be improved. The procedure involves invasive surgery, therefore, it is extremely important to select patients, so that those who undergo testicular tissue cryopreservation are more likely to benefit from future applications, in addition, patients who need gonadotoxic therapy are at an additional risk of bleeding and infection, particularly those with hematologic disorders such as leukemia or aplastic anemia (118, 125–127).

### 3.2 Sperm cryopreservation

Sperm cryopreservation is an effective method used in the management and preservation of fertility of animals and humans through assisted reproduction techniques (ART) (128, 129). This technique is based on the freezing of sperm to maintain its viability and functionality, and when performed correctly, it allows long-term freezing, as it results in the arrest of cellular metabolism that prevents cellular aging, maintaining viability and fertilization potential, an essential part for ART (130). The preservation of spermatozoa by freezing has the first record in 1776, but it was only in 1949 that the cryopreservation technique had its scientific progress with the discovery of the cryoprotective properties of glycerol (131), this advance being a point of departure within the field of fertility preservation (132). From this advance, there were significant improvements in the cryopreservation of semen of several species (133) with the creation of sperm cryobanks that took place during the 1960s for bovine species and in the 1970s for humans, the constitution of genetic resource banks began (134).

There are several conventional methods available for cryopreservation of human and animal semen: slow, fast and ultra-rapid freezing (known as kinetic vitrification) (135). The slow freezing method consists of progressive cooling divided into two or three stages over a period of 2 to 4 hours (136). In the first stage, sperm collected by ejaculation or other techniques is kept at room temperature for 10 minutes (137). In the second step, there is a gradual cooling of the samples from a temperature of 20°C to 5°C with a cooling rate of 0.5-1°C/min. After reaching a temperature of 5°C the samples are cooled again from 5°C to -80°C at a rate of 1-10°C/min, and finally, in the third step, the samples are frozen in liquid nitrogen (138). However, slow freezing leads to the formation of ice crystals resulting in high concentrations of electrolytes inside the cell, causing physicochemical damage to spermatozoa (139).

The rapid freezing method is based on the direct contact of samples with liquid nitrogen vapor for at least 10 minutes, in this method the sperm are mixed with cryoprotective agent and placed in cryotubes that will be exposed to nitrogen vapors. After the vapor exposure phase; the samples are immersed in liquid nitrogen (140, 141). The addition of CPAs to the samples seeks to minimize osmotic damage and prevent intracellular and extracellular ice crystals from forming, however, CPAs have cytotoxic characteristics (142).

Vitrification is the process of solidifying a liquid substance at extremely high freezing rates, transforming the liquid sample into an amorphous solid state (143). This freezing process prevents the formation of ice crystals (144). In addition, unlike the slow and fast methods, during vitrification, CPAs use is eliminated, as this method is cryoprotectant free (145). When used as a cryopreservation method, vitrification is commonly used for oocytes and embryos (146), since sperm vitrification is still a challenge due to the greater osmotic fragility of sperm when compared to other reproductive tissues (147).

Sperm cryopreservation is a tool for fertility preservation, sought by men who wish to start ARTs, in some cases being the only opportunity for couples to have children in the future (148). In addition, it is considered before starting any medical procedure that may affect male fertility, as in the case of non-malignant and malignant diseases, where it is necessary to submit the patient to chemotherapy, local radiology or radical testicular surgery (138). This technique is also recommended for men who have had a vasectomy. More recently, transgender patients, who chose to save their gametes for later use (149), have used cryopreservation. When used in animal species, cryopreservation is used for artificial insemination by making use of frozen and thawed sperm to improve rates of genetic improvement (150). It is considered a valuable tool, as it allows the preservation of genetic material from endangered species, through the storage of these gametes in cryobanks (5, 151).

Despite the high success rates in fertilization using cryopreserved semen, the technique has limitations such as intracellular and extracellular ice formation, osmotic and oxidative stress and toxicity from the use of cryoprotectants. These factors are responsible for cellular damage in the cryopreservation and thawing process (93, 152). Damage occurs to a greater degree during thawing, causing an imbalance in reactive oxygen species that directly affects cell metabolism and signaling, as well as DNA integrity and plasma membrane function and integrity (153). Recent studies point out that non-coding RNA (ncRNA), chromatin remodeling, DNA methylation and post-translational histone modifications are among the epigenetic factors involved in gene expression that are affected by cryopreservation and the thawing process (21). Sperm motility is the morphological parameter most affected by cryopreservation and thawing, caused by mitochondrial damage, sperm tail deformities and sperm membrane alterations (140).

### 3.3 Spermatogonial stem cell transplantation

Germ cell transplantation is an innovative technique that began to be used in 1994. The technique consists of isolating spermatogonial stem cells (SSCs) from a donor animal of interest and transplanting these cells into the testis of the recipient animal. After transplantation, the transplanted germ cells will continue their development and form mature and fertile sperm bearing the genetic characteristics of the donor animal (154). In recent years, this technique has been applied to mammalian species, in order to understand the processes of spermatogenesis and the biological characteristics of stem cells (155). In addition to these applications, the technique has a high potential for use in research related to biotechnology, genetically modified animals, and preservation of genetic material from endangered species or animals of high economic interest (38).

The use of this technique demonstrated for the first time that germ cells could be transferred between species and between animals of the same species (65). When microinjection of donor germ cells is performed in the seminiferous tubules of infertile recipients, a part of the donor germ cells moves to the periphery of the seminiferous tubules, moving through the Sertoli cell junctions. Due to the absence of spermatogenesis in recipient males, donated germ cells can penetrate the epithelial layer of Sertoli cells and reach the basal lamina (84). Considering the importance of understanding the steps involved during the *in vitro* spermatogenesis process and seeking to establish a favorable environment for the development and maturation of SSCs, further studies are still needed to prove the efficiency of SSCT in animals of the same species and between animals of different species.

### 3.4 Testicular graft

Testicular tissue grafting is a technique that has been studied to restore fertility. When compared to other restoration methods, grafting has several advantages such as the SSCs remaining within their microenvironment (avoiding the need for isolation and cell expansion *in vitro*) and providing an *in vivo* environment for the complete proliferation, differentiation, and maturation of germ cells (156). The complete process of spermatogenesis by the testicular tissue graft technique, that is, by allografts and xenografts, showed promising results in non-human species such as mice (157), hamster (158, 159); rabbit (160); bovine (161); rhesus monkey (162); horse (163); cat (164); dog (119) and buffalo (165).

In 2019, the first female offspring were born using sperm from grafts from prepubertal rhesus macaques (32). Despite the great advance in non-human species, in humans, xenotransplantation of immature testicular tissue (ITT) with spermatogonial cells was not able to carry out complete spermatogenesis, not producing sperm (166). Autologous grafts in humans have not yet been reported, therefore, data on testicular tissue autotransplantation in animal species provide important knowledge for the future application of this technique in human fertility (167).

As with all reproductive technologies, testicular grafting also has disadvantages, for patients diagnosed with neoplasms, autograft presents the risk of reintroduction of residual malignant cells present in cryopreserved ITT fragments (168). Therefore, continuous research to overcome the limitations of testicular grafts and provide information for application in humans is essential so that in the future this technique provides chances of success for patients to have biological children (169).

## 4 Discussion

### 4.1 Bioengineering principles applied to reproduction

**Figure 3:**
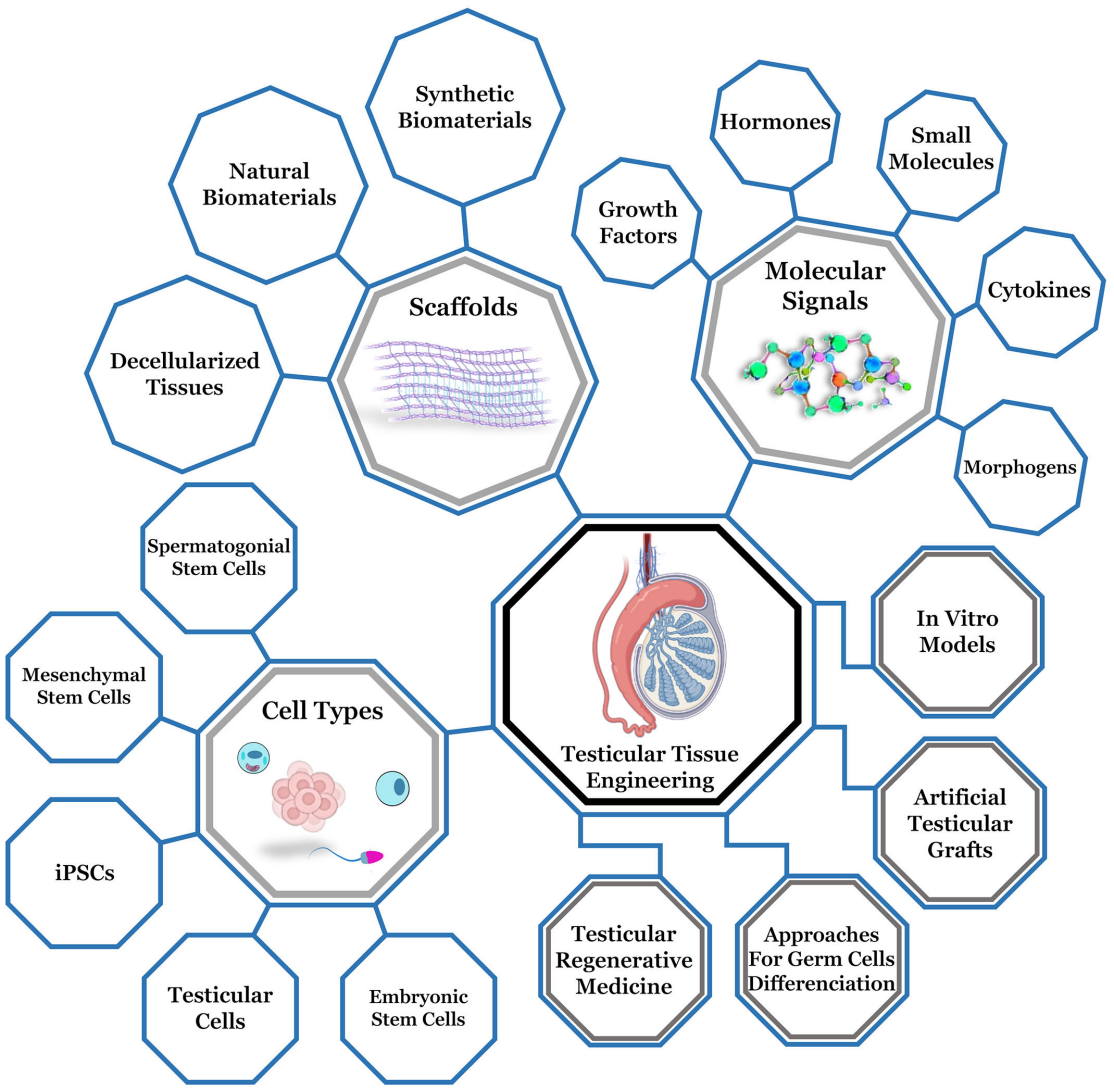
Representative diagram of testicular tissue engineering, highlighting the main components for testicular microenvironment reconstruction (biomaterials, cell types and molecular stimulation) and the main branches (testicular regenerative medicine, approaches for germ cells differentiation, development of artifical testicular grafts and production of *in vitro* models). Adapted from Biorender .

Conventional treatments for of male infertility and subfertility are still not effective related to problems associated to spermatogenesis disorders (170). Several testicular cell culture systems have been studied to mimetize the testicular microenvironment and restore fertility. An emerging field is reproductive tissue engineering (RTE), which is based on the same principles applied for vital organs as the heart, lungs, kidneys, liver, and skin (171–173). However, RTE must, in addition to reestablishing tissue integrity, generate a suitable microenvironment for the development of germ cells (174). Male reproductive tissues are under daily endocrine stimulation, with the rise and fall of testosterone, present a complex microarchitecture that changes due to intense hormonal flow (175). Aiming to mimetize these elements and testicular ECM, several biotechnological tools have been used to reconstruct testicular tissue and carry out the process of spermatogenesis *in vitro* (173) (**Figure 3**).

The understanding of the mechanisms involved during reproductive system development and how reproductive diseases occur has been of great value to develop *in vitro* models of reproductive tissues (41, 176). The advance of bioengineering has allowed the study of the male reproductive system in an innovative way by introducing new biomaterials that simulate organs and tissues that make up this system (177), by making bioprinted models (178–180). The microfluids (181) and biogels (58) production also may assist on cell development. The insertion of three-dimensional cell culture methods (182), maintaining physical and biochemical contact between cells and the tissue architecture brought the *in vitro* reality even closer to *in vivo*.

Studies report that cell-cell interaction in 3D culture systems influences the regulation and maturation of germ cells (183–186). 3D cultures favor the bidirectional communication between spermatozoa and the somatic cells that surround them, which are required for a proper testicular functioning and development (1, 187).

One of the limitations of *in vitro* spermatogenesis biotechnologies is to mimitize the testicular tissue microenvironment *in vitro*, as the interaction of gametes with the extracellular environment provides the necessary conditions to remain viable (188, 189). In search of alternatives to reproduce physiological conditions *in vivo* in the laboratory environment, tissue engineering approaches have been used to produce biomaterials that can reconstruct the structure of testicular tissue or assist in the process of spermatogenesis *in vitro* (190). Natural polymers such as collagen, fibrin and alginate have been explored because they are biomaterials with known biocompatibility and biodegradability (191). Synthetic polymers have also been studied to recompose the structural substrate for the maintenance of spermatogenesis, such as polyethylene glycol (PEG) and poly (epsilon-caprolactone) (PCL) (180, 192). Such materials can be chemically altered to adapt to certain conditions, however, as they are of synthetic origin, there are limitations in more accurately mimicking the extracellular environment (188).

Therefore, several synthetic and natural materials have been developed as extracellular matrix scaffolds, biogels, biodegradable polymers of gelatin, fibrin, collagen, hyaluronic acid, and poly (lactic-co-glycolic acid) that are widely used in tissue engineering (174, 193, 194).

#### 4.1.1 Classification of biomaterials in male reproductive tissue engineering

Biomaterials applied for reproductive tissue engineering can be divided in synthetic polymers, natural polymers (scaffolds and biogels) and decellularized matrixes. The main experimental approaches in synthetic and natural polymers are summarized in **Table 3**. Some studies aimed to obtain efficient decellularized tissues to be used as three- dimensional platforms to reconstruct an *in vitro* testicular microenvironment, which may allow several applications in reproduction medicine (**Table 4**).

**Table 3:** Studies using synthetic and natural biomaterials in testicular reconstruction approaches.

Biomaterial	Species involved	Type of study *(in vitro/in vivo)*	Types of cells used	Main biological findings	Reference
**Polylactic acid nanofiber (Poly- lactic)**	Mouse	*In vitro*	Spermatogonial Stem Cells	Study points that when using PLLA, there was a significant increase in the formation of spermatogonic cell clusters *in vitro*, compared to the control group cultured in a plate.	(195)
**Agar nanofiber/Poly (vinyl alcohol) (PVA)**	Mouse	*In vitro*	Spermatogonial Stem Cell	The combination of agar/PVA scaffold and growth factor-supplemented medium synergistically increased the differentiation rate of mouse SSCs into meiotic and post-meiotic cells. Thus, agar/PVA nanofiber scaffolds may have potential applications in infertility restoration, especially in azoospermic males.	(193)
**Collagen-based hydrogels**	Newt	*In vitro*	Spermatogonial Stem Cell + Sertoli cells	In this culture system, the differentiation of germ cells into primary spermatocytes occurred.	(196)
**Collagen hydrogels/collagen + Matrigel**	Rat	*In vitro*	Testicular cells isolated from seminiferous tubules (18 days after birth)	When cultivated *in vitro* in a 3D system using collagen gel matrix, the system provided increased viability, mitotic and mitotic division. Germ cells differentiate into spermatids.	(197)
**Collagen-based hydrogels**	Human	*In vitro*	Spermatogonial Stem Cell (nonobstructive azoospermia premeiotic or early meiotic maturation arrest)	In 3D culture in collagen gel matrix, spermatocytes were induced to differentiate into spermatids *in vitro*.	(198)
**Collagen-based hydrogels**	Mouse	*In vitro*	Spermatogonial Stem Cell + somatic testicular cells (7 dpp)	Collagen gel cultured with somatic testicular cells created a microenvironment similar to the seminiferous epithelium, which induced the process of spermatogenesis *in vitro*.	(199)
**Agarose gel**	Rat	*In vitro*	Testicular cells isolated from neonatal testis (7 dpp)	Three-dimensional cultures of mouse cell types influenced the functionality of Leydig cells, however, it did not influence the differentiation of germ cells, which can be explained by the lack of adequate organization of Sertoli cells.	(200)
**Alginate and fibrin hydrogels loaded with VEGF-NPs**	Mouse	*In vitro*	Testicular tissue of male NMRI mice (4–5 weeks)	VEGF-NPs encapsulated in alginate and fibrin hydrogel showed an increase in vascular density. Results obtained indicated that the alginate hydrogel preserved the spermatogonia, demonstrating a high rate of recovery after transplantation of avascular testicular tissue.	(201)
**Alginate hydrogel**	Mouse	*In vitro e in vivo*	Spermatogonial stem cells (6-day-old)	When injecting lyophilized spermatogonial stem cells encapsulated in an alginate-based hydrogel, spermatogenesis was recovered. By mimicking the cellular matrices, alginate supports the stemness provoked during the cellular cryopreservation process, restarting spermatogenesis after transplantation.	(202)
**Matrigel^®^**	Rat	*In vitro*	Testicular cells (18-day-old)	The culture model developed has organizational and functional similarities with the seminiferous epithelium in rat testis. Acquiring potential use for the cultivation of testicular cells *in vitro*.	(203)
**Fibrin**	Human	*In vitro*	Endometrial stem cells (hEnSCs)	Scaffolds containing human serum albumin (HSA)/tri calcium phosphate nanoparticles are easily produced and do not show cytotoxicity to spermatogonial stem cells.	(204)
**Chitosan-based hydrogel**	Human and rat	*In vitro*	Testicular tissue human (25 and 31 years of age) Rat (8- or 20-day-old)	The complete process of spermatogenesis was achieved both *in vitro* and *in vivo*. The culture system was defined using a bioreactor made of a hollow cylinder of a chitosan hydrogel that simulates the seminiferous tubules.	(194)
**Agarose gel**	Human	*In vitro*	Testis fragments (12- to 19-week fetuses)	Using agarose hydrogel, haploid spermatids recombined during meiosis, showing an increase in genetic diversity. Additionally, haploid spermatids performed the fertilization of oocytes, resulting in blastocyst formation.	(205)
**Agarose gel**	Mouse and Human	*In vitro*	Testis fragments (4 week-old)/spermatogonial stem cells	In three-dimensional testicular tissue culture, freezing SSCs slowly can induce the production of haploid cells.	(206)
**Agarose gel**	Mouse	*In vitro*	Testicular cells (2- to 6-day-old)	When cultured in agarose gel, testicular cells aggregated and performed spermatogenesis. By providing a suitable microenvironment, the cells differentiated to form morphologically mature sperm.	(207)
**Poly (D,L-lactic-co-glycolic acid) (PLGA)**	Rat	*In vitro*	Testicular cells	Rat testicular cells were cultured on the surface of the PLGA scaffold. It was observed that the scaffolds improved the proliferation and differentiation of germ cells in spermatogonia.	(208)
**Three-layer gradient system (3-LGS) using Matrigel^®^**	Rat	*In vitro*	Primary testicular cells	Using the three-layer gradient system (3-LGS), primary testicular cells were placed between two layers of cell-free Matrigel, such conformation creates a cell gradient that allowed the reorganization of testicular cells into organized structures.	(209)
**Matrigel^®^**	Mouse	*In vitro*	Testicular cells	The encapsulation of mouse testicular cells was carried out by Matrigel, the results showed that the cells self-organized into seminiferous tubules forming a blood-testis barrier (BTB), also promoting the differentiation of Leydig cells.	(210)
**Tri-calcium phosphate NPs + human serum albumin**	Mouse	*In vitro*	Spermatogonial Stem Cells	The scaffolds produced did not demonstrate cytotoxicity for the *in vitro* culture of SSCs.	(204)
**Calcium alginate**	Bull	*In vitro*	Testicular cells	Dissociation, reassembly and encapsulation of Sertoli cells and germ cells can improve long-term culture conditions so that germ cell differentiation could be realized.	(211)
**Nanofibrous scaffolds of poly L-lactic acid (PLLA)**	Mouse	*In vitro*	Spermatogonial Stem Cells	The presence of GDNF and BMP4 together with an antioxidant combined with electrically conductive 3D PLLA/MWCNTs fibrous scaffolds, and the presence of somatic cells in the culture are likely to build a testis-like microenvironment that promotes the growth and differentiation of SSCs.	(212)
**Poly L-lactic acid (PLLA)**	Mouse	*In vitro*	Spermatogonial Stem cells	Seeding of spermatogonial cells in PLLA may enhance the *in vitro* cluster formation of spermatogonial cells.	(213)
**Poly-l-lactic acid (PLLA)**	Rat	*In vivo*	–	When associated with PLLA scaffolds, spermatogenesis was significantly al.	(214)
**PCL/Gelatin nanofibrous scaffolds**	Human	*In vitro*	Spermatogonial Stem cells	The planned scaffold provided a suitable self-renewal microenvironment for the spermatogonial stem cells. The scaffolds produced have potential application in research and reconstructive medicine related to the field of male infertility.	(180)
**PCL/Gel Nanofibers**	Mouse	*In vitro*	Mouse Spermatogonial Stem Cells	The generated scaffolds were able to differentiate spermatogonial stem cells into spermatids.	(215)
**Alginate-based hydrogel**	Rat	*In vitro*	Spermatogonial cells (3–7 day-old)	This study is the first to report IVS in testicular constructs created by seeding single cell suspensions onto 3D bioprinted CFS and CLS.	(216)

**Table 4:** Decellularization studies to generate scaffolds for testicular bioengineering.

Biomaterial	Protocol of decellulariz ation	Species involved	Type of study (*in vitro/in vivo*)	Type of Cells Used	Main biological findings	Reference
**Decellularized scaffolds**	Via infusion of 0.5% sodium dodecyl sulfate (SDS) for 48 h, followed by 1% Triton X-100 for 6 h and then 1% DNase I for 1 h	Rat	*In vitro*	Testicular cells	The results revealed that the testis were successfully decellularized while maintaining the three-dimensional structure of the matrix and preserving the extracellular matrix components. After recellularization, the scaffold demonstrated that it supports cell adhesion and proliferation.	(217)
**Decellularized scaffolds**	0.5% sodium dodecyl sulfate (SDS) and 0.5% (v/v) Triton X-100 were applied for 2 h.	Mouse	*In vitro*	Spermatogonial Stem cells	After decellularization, the three-dimensional structure and constituents of the ECM remained preserved. The scaffold was successfully recellularized and had good cytocompatibility. *In vivo* tests showed some specific testicular cells, such as inhibin-positive cells within the scaffolds. In addition, the scaffold provided a microenvironment for DAZL-positive cell migration.	(218)
**Decellularized scaffolds**	Freeze-thaw cycle. After 1% Triton X-100 through the vas deferens for 4 h, 1% SDS for 48 h and 1% DNase for 2 h.	Rat	*In vitro e in vivo*	Mesenchymal stem cells collected from adult mouse bone marrow	After decellularization, the three-dimensional structure and constituents of the ECM remained preserved. The scaffold was successfully recellularized and had good cytocompatibility, *in vivo* tests showed some specific testicular cells, such as inhibin-positive cells within the scaffolds. In addition, the scaffold provided a microenvironment for DAZL-positive cell migration.	(60)
**Decellularized scaffolds**	0.01% sodium dodecyl sulfate for 7 h followed by 1 h of agitation in 1% Triton X-100	Swine	*In vitro*	Testicular cell organoids	Testicular cell suspensions isolated from immature porcine testicular tissue can form testicular organoids with seminiferous tubule organization comparable to the native organ when cultured *in vitro* in hydrogels. Testicular organoids showed somatic cell functionalities that were maintained until the end of the culture.	(43)
**Decellularized scaffolds**	The slices were decellularized in 1% sodium dodecyl sulfate (SDS) and then incubated for 24h.	Ram	*In vitro*	Spermatogonial Stem cells	The three-dimensional culture of SSCs in decellularized extracellular matrix provided adequate conditions for their preservation and proliferation. The results of this study may be a way to deepen the study of the process of spermatogenesis *in vitro*, as well as a hope for the treatment of infertility in men.	(219)
**Decellularized scaffolds**	1% Triton X-100 and/or 1% sodium dodecyl sulfate (SDS) for 24 or 48 hours.	Human	*In vitro*	Neonatal testicular cells	The scaffolds obtained after decellularization are not cytotoxic, providing adequate conditions that support the fixation and infiltration of testicular cells.	(220)
**Decellularized scaffolds**	Concentrations of 1%, 0.1% and 0.01% SDS were tested in the SDS-Triton (ST) and Triton-SDS-Triton (TST) protocols.	Swine	*In vitro*	Human primary Sertoli cells	The conditions of 0.1% and 3% TET offered the best decellularization in terms of DNA elimination and extracellular matrix (ECM) preservation, ensuring good fixation, proliferation and functionality of human Sertoli cells.	(221)
**Decellularized scaffolds**	0.5% (v/v) sodium dodecyl sulfate diluted in distilled water for 18 hours + 0.5% (v/v) Triton X-100 diluted in distilled water for 18 hours.	Mouse	*In vitro*	Spermatogonial Stem cells	Treatment of the mice’s whole testis with Triton X-100 and SDS efficiently removed the cells from the testis, so it is an appropriate protocol for the decellularization of whole testis.	(222)
**Decellularized scaffolds**	SDS	Rat	*In vitro*	Embryoid bodies	Recellularized testicular ECM may be a promissing tool for future new approaches for testicular cell differentiation applied for assisted reproduction techniques and infertility treatments	(223)
**Decellularized scaffolds**	100μm slices were decellularized with 1% SDS immersed for 24 hours.	Ram	*In vitro*	Spermatogonial stem cells	The results of the present study indicated that testicular scaffolds provide adequate conditions for the differentiation of SSCs.	(224)
**Decellularized scaffolds**	1% sodium dodecyl sulfate (SDS) in PBS solution for 18 h.	Mouse	*In vitro*	Mouse spermatogonial stem cells	The hydrogel scaffold containing 10 mg/ml decellularized ECM maintained the properties of SSCs at the molecular and cellular levels and promoted the differentiation of SSCs into round spermatids in the absence of somatic cells.	(58)
**Decellularized scaffolds**	Sodium dodecyl sulfate (SDS) 0.5%, 1%, 2%, Trypsin-EDTA 0.5%, 1%, Triton X-100 1% and 2%, respectively.	Ram	–	–	The 1% SDS perfusion protocol for 6-8 hours generated an acellular scaffold maintaining the integrity of the vascular network and preserving the three-dimensional structure as well as the extracellular matrix components.	(225)
**Decellularized scaffolds**	Hypertonic tris-buffer (TBS), 50 mM Tris-HCl pH 7.6 for 30 min, followed by 0.1% Triton X-100 for 15 min.	Ram	*In vitro*	Mouse spermatogonial stem cells	When cultured in acellular scaffolds, neonatal testicular cells from mice produced morphologically mature sperm in the shortest possible time. The scaffolds provided a microenvironment that functionally supported testicular cells, which secreted testosterone and inhibin B.	(179)
**Decellularized scaffolds**	1: 0.1% SDS for 24 hours 2: 0.5% SDS for 24 hours. 3: 1% SDS for 24 hours. 4: 0.5% SDS for 18 hours, then washed with PBS and immersed in 0.5% Triton for 18 hours.	Rat	*In vitro*	Spermatogonial cells	Immersion of testis from adult mice in 0.5% SDS solution and 0.5% triton solution was an effective method for decellularization of whole testicles without damaging the seminiferous tubules. The decellularized testicular scaffolds were biocompatible and had no detrimental effect on the viability of spermatogonial cells. Generated scaffolds supported spermatogonial cell proliferation during two weeks of culture.	(226)
**Decellularized scaffolds**	0.5% (v/v) Sodium dodecyl sulfate, then in 0.5% (v/v) Triton X-100, for 18 hours.	Rat	*In vitro*	Spermatogonial stem cells (6-day-old)	Spermatogonial stem cells can proliferate and differentiate into spermatocytes after being injected into decellularized testicular structures.	(62)
**Decellularized scaffolds**	Sodium hypochlorite solution 1.25%	Human	*In vitro e in vivo*	Induced Pluripotent Stem cells (iPS)	A 3D cell culture model was developed to generate human male germ cells from iPSCs and this model was compared to conventional 2D culture. Considering the effect of the 3D scaffold in the induction of specific markers of male germ cells, an increase in the efficiency of germ cell differentiation was observed.	(1)

##### 4.1.1.1 Synthetic polymers in testicular bioengineering

The use of synthetic polymers in tissue bioengineering is indicated because such polymers are biocompatible, have suitable physical-mechanical properties and elasticity to produce scaffolds. Among the synthetic materials used for the composition of scaffolds, there are the poly (L-lactic acid) (PLLA) (195, 213, 214), polypropylene, poly D, polycaprolactone (PCL) (215) and L-lactic-co-glycolic acid (208), which have already been used in the production of bone (227), cartilaginous (228), vascular (229) and dermal (230) scaffolds. Synthetic polymers can be combined with natural polymers, which improves their mechanical, physical and biocompatibility qualities.

However, even with favorable characteristics, few studies use synthetic polymers in male reproductive bioengineering. Among the studies found, Lee et al. (2011) (208) evaluated the ability of immature murine testicular cells to perform spermatogenesis *in vitro* when cultivated in biodegradable microporous scaffolds based on poly (D,L-lactic-co-glycolic acid) (PLGA). The results demonstrated that PLGA scaffold appears to provide a favorable microenvironment for spermatogenic germ cells to proliferate and differentiate into mature spermatids.

In the study of *in vitro* spermatogenesis, Ghorbani et al. (2019) (212) and Tseng et al. (2022) (214) used PLLA to produce scaffolds that were later cultured with spermatogonial cells. The results found showed that the use of PLLA in the manufacture of scaffolds could create a microenvironment like the native testis that promotes the growth and differentiation of spermatogonial stem cells.

The synthetic polymer polycaprolactone (PCL) was also used to study the proliferation and differentiation of spermatogonial stem cells, and the scaffolds produced from PCL were able to promote the expansion and differentiation of SSCs into spermatids (179). Thus, further studies are required to evaluate the advantages of synthetic polymers for the development of spermatogenesis *in vitro* models, since the results already obtained demonstrate that hydrogels based on synthetic polymers provide a favorable environment for germ cells to proliferate and differentiate into mature spermatids.

##### 4.1.1.2 Natural biomaterials for testicular bioengineering

Natural polymers are often used in tissue engineering because they have functional properties like those of natural ECM, presenting characteristics that assist on cell behavior, cell adhesion, migration, and differentiation (231). Unlike synthetic polymers, natural polymers are more biodegradable and biocompatible and are widely used in male reproductive tissue engineering (232).

The main natural polymers used are alginate (211, 216), fibrin (201, 204), collagen (196, 233), Matrigel (203, 209, 210, 234), agarose (200, 205–207, 235) that are used as scaffolds to build a testicular microenvironment *in vitro*. The use of hydrogels has been the main method to reconstruct a three-dimensional testicular microenvironment, as it increases the contact surface between the material and the cells (236).

Several studies have used alginate for encapsulation of spermatogonial stem cells due to its cell compatibility, gelling property, biodegradability, and mechanical strength (237). When investigating the biocompatibility of alginate in the encapsulation of mouse spermatogonial stem cells, Jalayeri et al. (2017) (238) found that alginate hydrogel is a non-toxic compound that does not affect the viability and morphology of stem cells and can be used in the encapsulation of spermatogonial stem cells. Veisi et al. (2022) (239) used co-cultured spermatogonial stem cells encapsulated in alginate hydrogel with Sertoli cells and found that culturing SSCs in alginate hydrogel with Sertoli cells in a 3D culture can lead to efficient proliferation and maintenance of SSC and increase the efficiency of SSC transplantation. Poels et al. (2016) (201) evaluated two different compositions of hydrogels, one made of 1% alginate and the other made of fibrin (30 mg/mL fibrinogen/30 IU/mL thrombin), the results demonstrated an improvement in the survival of the spermatogonial subpopulation with the use of alginate matrix compared to fibrin gel.

Some studies have used fibrin as a scaffold for testicular reconstruction since this polymer assisted on wound healing and tissue regeneration by having bioactive factors such as fibronectin that act as a substrate for cell migration and anchorage (240). Ramzgouyan et al. (2015) (241) were able to differentiate germ cell-like cells on fibrin hydrogel, demonstrating the biocompatibility of the differentiated cells to the hydrogel. Although little studied, fibrin scaffolds have high porosity and biocompatibility, characteristics that can be used for the development of seminiferous tubule niches in testicular tissue (170).

Hydrogels based on collagen fibers have been used to develop methodologies for the cultivation and differentiation of male germ cells (198, 199, 242, 243). Studies using 3D structures based on collagen hydrogels report promising results regarding differentiation (242), maturation (198), maintenance of testicular cell viability (244) and provided support for *in vitro* spermatogenesis (199). The differentiation of spermatogonial cells in culture medium supplemented with collagen-based hydrogel is also pointed out (243).

Used as a support for the development of germ cells *in vitro*, Matrigel is a natural polymer derived from ECM, having several essential components such as laminin, collagen, and fibronectin. Its use in the cultivation of cells and tissues *in vitro* is due to its promoting effects on cell growth and differentiation (174). Matrigel has been used to differentiate functional haploid spermatids in previous studies. Sun et al. (2018) (234) reported that a three-dimensional induced system with Matrigel differentiated human SSCs into functional haploid spermatids. To evaluate differentiation, cell content and meiotic chromatin scattering assays were performed, which revealed that spermatocytes and haploid cells were effectively generated from human SSCs by the three-dimensional induced system with Matrigel. Another study reported the creation of a three-layer gradient system being one layer of Matrigel with testicular cells in the middle of two layers of Matrigel. The results demonstrated that testicular cells migrated within the Matrigel, forming testicular organoids with growing germ cells and presented a functional blood-testis barrier (245).

##### 4.1.1.3 Decellularized ECM for testicular microenvironment reconstruction

Biological scaffolds from the decellularization process must present some characteristics to be considered ideal, such as: absence of toxicity, non-immunogenicity, non-pathogenicity and be biodegradable to allow cell adhesion and provide appropriate conditions for the creation of a biological scaffold microenvironment that can carry out cell growth, proliferation, and migration (77, 222). After the decellularization process, the bioactivity of growth factors present in acellular scaffolds such as VEGF, TGF-β and bFGF remains unchanged (246).

Several decellularization protocols reported range from chemical to enzymatic methods, depending on the type of tissue and its biological properties (220, 221). Obtaining decellularized scaffolds is commonly done by the agitation technique, however, recent studies have been using the technique of perfusion of cellular detergents *via* arterial route to break cellular bonds with the ECM and dissolve cellular materials and their debris from within the tissue or organ (225, 247). Among the most used detergents and solutions are sodium dodecyl sulfate (SDS), ethylenediaminetetraacetic acid (EDTA), sodium hypochlorite and Triton (X-100) (58).

It is known that ECM acts in the spermatogenesis process, through laminin and collagens that allow the differentiation of germ cells, promoting their change from the basal lamina to the lumen of the seminiferous tubules, through adjustments of the structural junction (66). Thus, the creation of a testicular scaffold from ECM that can support testicular cells may provide new information about the fundamental cell-matrix interactions that occur during spermatogenesis (64).

The interaction of male gametes with the microenvironment is essential for sperm development in testicular tissue (188, 218). The extracellular matrix is ​​a major player in this process, as the communication and molecular signaling of the components of the ECM with the spermatozoon not only provides structural support to the gamete, but also plays an active role in maintaining its viability, as well as in the transport and distribution of essential substances to the gamete (248).

Decellularized extracellular matrix scaffolds can be biotransformed into biogels through solubilization followed by neutralization and gelation (249). Although the production of biogels from decellularized ECM scaffolds alters the three-dimensional conformation of the matrix, biogels can retain bioactive factors present in the ECM of native tissues, preserving the ability to guide and favor specific cellular behaviors through orientation by contact, and by fibrillation of proteins that interact with integrin receptors on the cell membrane (250, 251). In relation to scaffolds, biogels have some advantages, as they require the use of less invasive procedures for their deposition at the target site and facilitate the repair of irregular tissue surfaces due to their viscoelasticity, provided by collagen and other biomolecules that compose them (252). Thus, in addition to the new possibilities of cell culture to improve *in vitro* spermatogenesis rates, bioengineering proposes innovative methods of treatment for diseases that affect the reproductive system and the resumption of fertility, such as the application of decellularized matrix scaffolds or biogels (217, 253).

The reciprocal interactions performed by Sertoli cells, Leydig cells, germ cells, testicular endothelial cells, peritubular myoid cells and macrophages in the microenvironment in which they are found are of fundamental importance for the proper process of spermatogenesis (254). Various components of this microenvironment (e.g., growth factors, cytokines, hormones, and adjacent cells) and the way they orchestrate cellular development have been the subject of a lot of research over the last few years (255). On the other hand, the extracellular matrix that composes these tissues remained little explored, being recognized only as a passive component of cellular anchorage (256). With the discovery of the physicochemical properties of the extracellular matrix (ECM) of various organs, the hypothesis that this component plays the role of a simple cellular framework was abandoned, going from a mere protagonist to an active modulator of cellular functions that govern morphogenesis and tissue repair (257).

### 4.2 Techniques designed to preserve male fertility

#### 4.2.1 Application of hydrogels in restoring male fertility

Providing a transitory tissue-mimicking environment for cell proliferation and differentiation to occur, hydrogels have high structural similarity to ECM, being used to encapsulate cells and tissues within reproductive bioengineering. Its composition is mostly water (90%), allowing the diffusion of nutrients, providing physical support for the cells (173). Recently, several studied applied hydrogels in testicular tissue and in cell culture focusing on the differentiation of spermatogonial stem cells into haploid spermatozoa (258). Hydrogels can be produced from natural and synthetic polymers; however, hydrogels from natural polymers are more used for their non-toxic properties and biocompatibility (259). As they have biodegradable and bioresorbable properties, these materials will provide functional 3D matrices for cells and tissues without inducing inflammation (260). Considering that the ECM is mainly composed of proteins and polysaccharides (257), two groups of polymers have been used for the spatial arrangement of testicular cells: proteins as collagen and polysaccharides such as chitosan and alginate (197).

As the most abundant structural protein, collagen is the most investigated polymer in the production of hydrogels to restore male fertility (199). Hydrogels produced from collagen provide a favorable environment for the differentiation and maturation of germ cells, offering a niche for the reassembly of testicular cells isolated from animals and humans (261). The use of collagen hydrogels alone or in combination with Matrigel allows germ cells to be in contact and actively interact with somatic cells and the ECM (262, 263). In a study by Lee et al. (2006) (242), rat testicular cells were cultured in collagen gel and collagen gel + Matrigel. The results showed that the matrices used showed the potential for reassembly of dissociated cells, provided meiotic support, post-meiotic progression and differentiated male germ cells.

Hydrogels are also being used to encapsulate spermatogonial stem cells during the cryopreservation process. Pirnia et al. (2017) (202) used alginate-based hydrogel for encapsulation of mouse spermatogonial stem cells during the cryopreservation process. The group performed a comparison of the colonization potential and degree of viability of SSCs before and after the freeze-thaw cycles. The results demonstrated that there were no differences in the freeze-thaw cycles, and after thawing there was a successful restoration of spermatogenesis.

#### 4.2.2 Acellular ECM scaffolds

Biological scaffolds are generated by the process of tissue decellularization, which can occur both in the entire organ and in part of it (226, 264). These scaffolds act as the architecture for tissue formation and are normally seeded with cells and, occasionally, growth factors, or subjected to biophysical stimuli provided by a bioreactor, which consists of a device or system that applies different types of mechanical and chemical stimuli on the cell culture (265).

Therefore, the material that was previously decellularized is considered and used as a biological scaffold. After being inserted *in vivo*, the scaffolds generated by tissue decellularization, have properties similar to those of physiological tissues and provide the structural basis for aggregation to adjacent tissues (225, 266).

Thus, the development of biological and biocompatible scaffolds can present benefits for *in vitro* germ cell culture systems, recently, the use of these scaffolds for the *in vitro* spermatogenesis process has been considered promising (62, 224). The extracellular matrix is ​​a tool to study disorders that affect spermatogenesis. Several biomaterials and scaffold manufacturing methods have been investigated for application in testicular tissue engineering (267). The development of scaffolds from biomaterials reduces problems associated with post-transplant complications, such as tissue deficiency and the use of immunosuppressive drugs.

The scaffold also provides a basis for performing tissue recellularization by culturing isolated cells *in vitro* or in the host *in vivo* by cell migration (223). Several types of stem cells can be used in the differentiation of germ cells, especially in cases of disturbances in spermatogonia (60).

Based on these characteristics, research has focused on optimizing protocols for decellularization of male reproductive tissues to obtain a scaffold that presents a three-dimensional structure that conforms to the components of the matrix preserved, thus enabling good development, adhesion, and cell proliferation in modalities of 3D cultivation (219, 262). Among the protocols that have been developed, the use of SDS and Triton X-100 at a concentration of 0.01% in the decellularization of immature swine testis fragments supported the cultivation of Sertoli cells. The data obtained showed that the scaffolds produced by the combination of two cellular detergents in low concentration preserved the functionality of vital testicular cells such as Leydig, peritubular, myoid cells and SSCs (221). Another study performed the cultivation of human Sertoli cells in decellularized ECM from porcine testis, pointing out that the use of decellularized porcine testis or testicular ECM from other animal species is viable in testicular bioengineering (60).

Recently, Vermeulen et al. (2019) (43) organized testicular organoids were generated in decellularized ECM-based hydrogels to restore male fertility. The system had an appropriate storage module (capacity to store energy in the elastic deformation of the material) for the porcine testicular organoid culture. The scaffold produced was able to form structures of the seminiferous tubule and showed that there was a preservation of growth factors within the organoids in addition to presenting regenerative capacity.

#### 4.2.3 Approaches without using scaffolds

Traditionally tissue engineering involves a scaffold, bioactive factors, and cells. Materials used as scaffolds affect cell behavior, influence in their growth, proliferation, and differentiation (268). Despite this great stimulation, these approaches require complex and expensive techniques. As an alternative, scaffold-free methods have emerged. Through cell self-assembly technique, scaffold-free methods generate 3D multicellular aggregates that secrete their own matrices (269).

*In vitro* models of human testicular organoids that perform characteristic functions of testis have already been reported (270). However, morphologically there is no similarity of these organoids with testicular tissue. The models that most efficiently mimetize the testicular architecture is the suspension culture models that allow the expansion of germ cells and the incorporation of somatic cells. Pendergraft et al. (2017) (270), in non-human primates, did not achieve the progression of spermatogenesis, only the expansion of spermatogonia, raising the hypothesis that the addition of factors such as physiological microenvironments is necessary.

Soluble human testis ECM have been employed as an additive medium for the cultivation of human testicular organoids to mimetize the testicular microenvironment without providing a structural scaffold (271).

#### 4.2.4 Microfluidic systems

In experiments using human materials, there are ethical and experimental limitations such as low availability of resources and difficulty in long-term *in vivo* maintenance of artificially produced tissues and organs. These limitations make *in vitro* human fertility research challenging. As an alternative to overcome such limitations, micro/nanofabrication techniques are being used, one of these techniques are microfluidics, which has the potential to promote a significant increase in techniques used in male reproduction clinics (272). The microfluidics technique is defined as the technology that designs, manufactures, and models devices for handling and analyzing small amounts of fluids (273).

With the use of microfluidic systems, male fertility restoration researchers can remove barriers that limit the results obtained, such as testicular cell death, limitation of primary testicular cells and the absence of new tools that can mimic the complexity of testicular tissue (99). Komeya et al. (2019) (274) cultured testicular tissue fragments from neonatal mice in a simple microfluidic device. The system was manufactured in a simple way and managed to maintain endocrine functions and spermatogenesis for 6 months. The microfluidic device used separated the testicular tissues and the fluid medium through a thin porous membrane while the culture medium flowed in channels that mimicked the capillaries.

In another experiment Kojima et al. (2018) (275), seeded neonatal mouse testis in agarose gel molded on a microfluidic chip. The polydimethylsiloxane (PDMS) present on the surface of the hydrogel showed high oxygen permeability and was able to support the transport of oxygen to the tissue layers, thus preventing central tissue necrosis and increasing cell growth during the 7 days of the experiment. In 2019, the group of Komeya et al. (2019) (274) carried out a follow-up work on the study cited above, where they placed the testicular tissues of immature mice in agarose gel blocks and forced the spread in monolayer through a microfluidic ceiling system. As a result, they observed that, when in the presence of the PMDS microfluidic device chip, the spermatogenesis process was initiated and maintained, followed by the increase in meiotic germ cells, the findings also demonstrated that the spermatogenesis process was able to differentiate the cells in the microfluidic system until the stage of rounded/elongated spermatids.

#### 4.2.5 Bioprinting and 3D printed scaffolds

Through 3D printing, the fabrication of materials used in reproductive tissue bioengineering occurs with precision of the spatial geometry and internal microarchitecture of the pores, enabling the creation of a personalized biomimetic environment (175). In this way, this technology allows the integration of several biomaterials and multiple cell varieties, generating a 3D biomimetic structure. In male reproductive biotechnology, 3D-printed alginate scaffolds have been studied for the generation of organoids, however, a biomimetic morphology like the native testis was not observed (216).

3D bioprinting consists of the deposition of biocompatible materials, cell types and support components by computer generating complex 3D tissues. For the manufacture of 3D scaffolds, several layers of biological materials, biochemical compounds and cells are printed in sequence, making it possible to spatially control the positioning of the components used in bioprinting (276). Research with the application of 3D bioprinted materials is in early stages, however clinical applications with the generation and transplantation of bones and skin have already been reported (277).

It is essential to obtain a 3D structure that can be applied in bioengineering that the mechanical, structural, and functional components present characteristics like tissues *in vivo* (278). Therefore, the chosen biomaterials should always be chosen considering biocompatibility, easy handling, easy printing and maintenance of cell viability and function (279). However, some challenges are faced during 3D bioprinting such as the correct choice of design, as well as the choice of materials, cell types and growth and differentiation factors (269).

Baert et al. (2019) (216) performed the 3D bioprinting of alginate-based scaffolds for the study of spermatogenesis *in vitro*. After the bioprinting of the scaffolds manufactured, the authors carried out the cultivation in the scaffolds with testicular cells and observed that at the end of the days of cultivation there was a differentiation to the level of round spermatids and elongated spermatids, suggesting that the model created may be useful for studies (duck) of physiological and drug screening applications. Another study that used 3D bioprinting for *in vitro* production of spermatozoa was by Bashiri et al., 2022 (179). During the experiment, decellularized extracellular matrix of ram testis was used in 3D bioprinting, and these scaffolds were later manufactured and cultivated with spermatogonial stem cells. The results showed that 3D-printed scaffolds derived from decellularized extracellular matrix increased the viability and proliferation of spermatogonial stem cells, through the release of growth factors.

## 5 Future perspectives

When performed *in vivo*, spermatogenesis is a complex process being controlled by the endocrine system and totally dependent on the testicular microenvironment. Looking for solutions for the restoration of male fertility, reproductive bioengineering has been developing and introducing into research various types of biomaterials, whether synthetic, natural or from extracellular matrix. The projection of biomaterials for studies of infertility and recovery of male fertility is important since the number of cases of male infertility in humans and animals is increasing. Due to the structural and functional complexity of the testis that require specific hormones to carry out spermatogenesis, biomaterials that can promote structural regeneration and functional recovery of male gonads have not yet been developed. Currently, these biomaterials present a great potential as a tool for male reproductive medicine in applications such as screening assays in drug development and toxicology due to their adaptable and scalable resources. These new approaches may become more viable and efficient to be used as alternatives for male fertility preservation and restoration in comparison to other systems as *in vitro* spermatogenesis, culture of organotypic fragments. Seeking to overcome the challenges of spermatogenesis *in vitro* and *in vivo*, several advanced technologies are being used in the biomanufacturing of biomaterials for use in reproductive bioengineering. Among the techniques being designed to preserve male fertility are hydrogels, decellularized extracellular matrices, techniques without the use of scaffolds, microfluidic systems and 3D scaffold bioprinting. 3D bioprinting with the printing of cells on the scaffolds produced is a technique that has been growing within the field of bioengineering, with the advantage of controlling the spatial deposition of biomaterials and cell types. In addition, the application of microfluidic devices, chip platforms and other technologies have been employed in the construction of reproductive organoids. In future studies, such technologies can be combined with biomaterials that are being manufactured to mimic the testicular microenvironment.

Understanding the influence that the extracellular matrix performs on the testicular microenvironment makes it possible to choose the best biomaterial to be used for testicular regeneration and in the study of spermatogenesis *in vitro*. Thus, the use of biomaterials discussed in testicular bioengineering can still replace the requirement for experimental animals in *in vitro* spermatogenesis research, providing biomaterials that mimetize the testicular microenvironment.

## Author contributions

Conceptualization, BiOH-P, LS, GA, AC and MM. Writing-original draft preparation, BiOH-P, LS, GA, Ba´OHPP, JF, MP and PD. Writing-review and editing, BiOH-P, Ba´OHPP, LS, GA, AC MM. Supervision, AC and MM. Funding acquisition, MM. All authors contributed to the article and approved the submitted version.
